# A vacuolar sorting receptor-independent sorting mechanism for storage vacuoles in soybean seeds

**DOI:** 10.1038/s41598-017-18697-w

**Published:** 2018-01-18

**Authors:** Nobuyuki Maruyama, Yuki Matsuoka, Kazunori Yokoyama, Kyoko Takagi, Tetsuya Yamada, Hisakazu Hasegawa, Teruhiko Terakawa, Masao Ishimoto

**Affiliations:** 1 Graduate School of Agriculture, Kyoto University, Gokasho, Uji, Kyoto, Japan; 2 National Institute of Agrobiological Sciences, Tsukuba, Ibaraki, Japan; 3 Graduate School of Agriculture, Hokkaido University, Kita-ku, Sapporo, Hokkaido, Japan; 4 Hokko Chemical Industry Co., LTD, Atsugi, Kanagawa Japan; 5 Present Address: Tohoku Agricultural Research Center, NARO, Fukushima, Japan; 6 Present Address: INPLANTA INNOVATIONS INC, Yokohama, Kanagawa Japan; 7 Present Address: Institute of Crop Science, NARO, Tsukuba, Ibaraki Japan

## Abstract

The seed storage proteins of soybean (*Glycine max*) are composed mainly of glycinin (11S globulin) and β-conglycinin (7S globulin). The subunits of glycinin (A1aB1b, A1bB2, A2B1a, A3B4, and A5A4B3) are synthesized as a single polypeptide precursor. These precursors are assembled into trimers with a random combination of subunits in the endoplasmic reticulum, and are sorted to the protein storage vacuoles. Proteins destined for transport to protein storage vacuoles possess a vacuolar sorting determinant, and in this regard, the A1aB1b subunit contains a C-terminal peptide that is sufficient for its sorting to protein storage vacuoles. The A3B4 subunit, however, lacks a corresponding C-terminal sorting determinant. In this study, we found that, unlike the A1aB1b subunit, the A3B4 subunit does not bind to previously reported vacuolar sorting receptors. Despite this difference, we observed that the A3B4 subunit is sorted to protein storage vacuoles in a transgenic soybean line expressing the A3B4 subunit of glycinin. These results indicate that a protein storage vacuolar sorting mechanism that functions independently of the known vacuolar sorting receptors in seeds might be present in soybean seeds.

## Introduction

Proteins destined for transport to protein storage vacuoles (PSVs) possess a vacuolar sorting determinant (VSD)^1^. The VSDs of seed storage proteins are categorized into three types: sequence-specific VSDs (ssVSDs), C-terminal VSDs (ctVSDs), and physical structure VSDs (psVSDs). The 2S albumin seed storage proteins of the Brazil nut (*Bertholletia excelsa*) and castor bean (*Ricinus communis*) contain ctVSDs and ssVSDs, respectively^2,3^. The four C-terminal residues of phaseolin (the 7S globulin of the common bean) are exposed on the molecular surface and function as ctVSDs^4^, whereas the 10 C-terminal residues of the α′ and β subunits of 7S globulin of soybean are essential for sorting to the PSV in transgenic *Arabidopsis* seeds and in maturing soybean cotyledons^5,6^. All types of VSDs have been reported in 11S globulins^7–9^. Legumin has a putative psVSD, although its exact character remains unclear^7^. The internal peptides of cruciferin, the *Arabidopsis thaliana* 12S globulin, are sufficient for vacuolar sorting^8^.

Vacuolar sorting receptors (VSRs) for VSDs are involved in plant vacuolar sorting^1^, among which are members of the BP-80 family of type I membrane proteins^10–12^. These VSRs are required for the transport of soluble proteins with VSDs from the endoplasmic reticulum (ER) to the trans-Golgi network in a transport pathway that involves the Golgi^13^. Although there is currently limited information available regarding soybean VSRs, there has been significant work on VSRs in the model plant *Arabidopsis thaliana*. In *A*. *thaliana*, there are seven genes for VSR (AtVSR1-7)^14^. In an AtVSR1 knockout mutant, the seed storage proteins 2S albumin and 12S globulin are partially secreted into the intercellular space in seeds^15^. Similarly, green fluorescent protein fused with the C-terminal peptide of the β-conglycinin α′ subunit is also secreted into the intracellular space in a AtVSR1 knockout mutant^16^. VSR classes, including AtVSR1, AtVSR3, and AtVSR4, are involved in trafficking to the vacuole^14^.

In soybean (*Glycine max*), these seed storage proteins are mainly composed of 11S globulin (glycinin) and 7S globulin (β-conglycinin), of which glycinin accounts for approximately 40% of total seed protein^17^. The five major glycinin subunits are classified into two groups according to their amino acid sequences (group I: A1aB1b, A1bB2, and A2B1a; group II: A3B4 and A5A4B3). These glycinin subunits are synthesized as single polypeptide precursors, and their signal sequences are removed co-translationally in the ER, wherein the resultant proglycinin assembles into trimers comprising a random combination of subunits, which are subsequently sorted to the PSVs. A specific posttranslational cleavage between Asn and Gly residues in the PSV produces an acidic and a basic polypeptide of the subunits, linked by a disulphide bond, with a hexamer formation.

In maturing soybean cotyledons, the C-terminal 10 residues of the A1aB1b subunit of glycinin are sufficient as a ctVSD for sorting to the PSV, whereas the A3B4 subunit lacks a C-terminal peptide corresponding to the ctVSD of the A1aB1b subunit, and is assumed to contain a VSD other than the ctVSD type^9^. Since endogenous glycinin forms hybrid molecules with expressed derivatives, it is difficult to examine sorting of the A3B4 subunit independently in transgenic soybean. In this regard, a soybean breeding line (the JQ line) lacking both glycinin and β-conglycinin, and with competency for transformation, embryogenesis, and regeneration, has been developed^18^. In the present study, we used the JQ line to investigate the sorting of the A3B4 subunit to the PSV in soybean. In addition, we analysed the interactions of soybean VSR with glycinin subunits. The results indicate the existence of a vacuolar sorting mechanism in soybean seeds that functions independently of the known receptors in seeds.

## Materials and Methods

### Construction of expression plasmids for the luminal domain of GmVSR

GmVSR cDNA was obtained by RT-PCR as previously reported^19^. The recombinant luminal domain of GmVSR [GmVSR(LU)] was prepared by using the BaculoDirect Baculovirus Expression system (Thermo Fisher Scientific, Waltham, MA, US) according to the manufacturer’s instructions, as previously described^20^. We amplified GmVSR(LU) using the primers 5′-AAGCTTCGGAGATCTTCGCTGTGCGTCTTTC-3′ and 5′-AATCCGCTCGAG**TCA***GTGGTGGTGGTGGTGGTG*TCTTCCCTCCTGACTGGCAGTTTTACTTATGCAAG-3′ (italic, underlined, and bold letters indicate a polyhistidine tag region, *Xho*I site, and stop codon, respectively). The amplified fragments were digested with *Xho*I and ligated with the *Nco*I (filled in)- *Xho*I-digested entry vector pENTR4 to produce the entry clone pENTRGmVSR, which encodes the signal sequence and the luminal region of GmVSR followed by the polyhistidine tag. The sequence of this plasmid was determined using an ABI 3100 Avant DNA Analyser (Thermo Fisher Scientific). Subsequently, to construct the recombinant baculovirus DNA, a recombination reaction was performed between each entry clone and BaculoDirect linear DNA using LR Clonase II Enzyme Mix (Thermo Fisher Scientific). *Spodoptera frugiperda* (Sf9) cells were then transfected with the recombinant baculovirus DNA using Cellfectin (Thermo Fisher Scientific). Five days after transfection, the cell culture medium was harvested and used as a P1 viral stock.

### Expression and purification of the recombinant GmVSR luminal domain

Optimal expression times for GmVSR(LU) were determined by monitoring cellular extracts using SDS-PAGE and immunoblotting with an anti-polyhistidine tag antibody. Three days after infection, the media were collected by centrifugation at 10,000 × *g* for 10 min, and thereafter incubated with Ni Sepharose FF (GE Healthcare Life Sciences, Little Chalfont, UK). After washing with a low salt buffer (20 mM HEPES-NaOH, pH 7.0, 150 mM NaCl, 0.4% CHAPS, and 1 mM CaCl_2_), GmVSR(LU) was eluted using a gradient of 20–500 mM imidazole in the low salt buffer. The fractions containing GmVSR(LU) were concentrated using Vivaspin 30 columns and were then loaded on a Hiload 16/600 Superdex 200 pg column (GE Healthcare Life Sciences) equilibrated with the low salt buffer.

### Purification of GST fused with the C-terminal peptide of the A1aB1b subunit

Expression plasmids for glutathione *S*-transferase (GST), GST fused with the 10-residue C-terminal peptide of A1aB1b (GST-A1aB1bCT10), and GST fused with the 10-residue C-terminal peptide of A1aB1b and an additional six glycine residues (GST-A1bB1bCT10 + 6 G) were constructed by PCR with pGEX6p-1 (GE Healthcare Life Sciences) as a template using the following primers: 5′-TCCCAGGGGCCCCTGGAACAGAACTTCCAG-3′ and 5′-TAGCCGCATCGTGACTGACTGACGATCTGCCTC-3′ for GST, 5′-AGCTCTCTTCTGAGACTCCTGAGGTCCCAGGGGCCCCTGGAACAGAAC G-3′ and 5′-GTGGCTTAGCCGCATCGTGACTGACTGACGATC-3′ for GST-A1aB1bCT10, and 5′-AGCTCTCTTCTGAGACTCCTGAGGTCCCAGGGGCCCCTGGAACAGAAC G-3′ and 5′-GTGGCTGGAGGAGGAGGAGGAGGATCGCCGCATCGTGACTGACTGACG-3′ for GST-A1bB1bCT10 + 6 G. *Escherichia coli* BL21(DE3) cells transformed with plasmids for individual GST proteins were grown to mid-log phase, and protein production was induced with 1 mM isopropyl-1-thio-β-d-galactopyranoside for 20–28 h at 25 °C. Cells were harvested, suspended in phosphate-buffered saline (PBS), and sonicated. Glutathione Sepharose 4B beads (GE Healthcare Life Sciences) were incubated with the supernatants overnight. After washing three times with PBS, the respective GST constructs were eluted from the resin with a Tris-HCl (pH 8.0) buffer containing 10 mM reduced glutathione.

### Expression and purification of recombinant A1aB1b and A3B4 subunits

The recombinant A1aB1b and A3B4 subunits were expressed and purified according to previously described methods with some modifications^21^. Cells expressing the recombinant A1aB1b and A3B4 subunits were harvested, resuspended, and sonicated in a potassium phosphate buffer (35 mM potassium phosphate, pH 7.6, 0.4 M NaCl). After centrifugation, the soluble proteins were applied to Ni Sepharose (GE Healthcare Life Sciences) resin to purify the recombinant A1aB1b and A3B4 subunits. For Ni Sepharose eluates containing the recombinant subunits, gel filtration chromatography was carried out using a Hiload 16/600 Superdex 200 pg column to obtain the recombinant A1aB1b and A3B4 subunits. An expression plasmid for A1aB1b fused with an additional six glycine residues at the C terminus (A1aB1b + 6 G) was constructed. The A1aB1b + 6 G protein was expressed and purified similarly to the recombinant A1aB1b or A3B4 subunit.

### Surface plasmon resonance and kinetic assays

GmVSR(LU) was immobilized on a BIACORE2000 (BIACORE, Tokyo, Japan) sensor chip. Carboxymethylated dextran on a CM5 sensor chip was activated with a solution containing 0.05 M *N*-hydroxysuccinimide and 0.05 M *N*-ethyl-*N*-(3-diethylaminopropyl)carbodiimide, and then coupled with GmVSR(LU). The amount of coupled protein on the sensor chip was between 1000 and 1200 resonance units.

The purified GST fusion proteins were injected onto the sensor chip for 180S, and then eluted with salt buffer for 180S at 25 °C at a flow rate of 30 μL/min. The sensor chip surface was regenerated with 90 μL of a chelating buffer (20 mM HEPES-NaOH, pH 7.0, 150 mM NaCl, 0.4% CHAPS, and 2.5 mM EGTA) to remove the residual GST fusion proteins on the sensor chip. Sensorgrams were generated by subtracting the sensorgram of the control flow cell.

Kinetic analysis was performed according to the manufacturer’s protocol. Kinetic constants [the association rate constant (ka), the dissociation rate constant (kd), and the dissociation constant (K_D_ = kd/ka)] were calculated from the sensorgrams using BIA evaluation software version 3.0 (BIACORE). The kinetic parameters were determined from three independent experiments.

### Preparations of transgenic soybeans

Transgenic soybean lines were prepared according to a method described previously^22^. For this purpose, we used a soybean breeding line (the JQ line) lacking both glycinin and β-conglycinin, and with high transformation efficiency^18^. A seed-specific Arcelin 5 promoter^23^ was used for expression of an A3B4 subunit construct in transgenic soybeans. The expression plasmid was transformed into soybean somatic embryos using the bombardment method^24^.

### Gel-filtration chromatography

Dry mature seeds were crushed into a powder using a multi-bead shocker (MB501S; YASUI KIKAI, Osaka, Japan). The resultant tissue powder was mixed with potassium phosphate buffer and vortexed at room temperature. The mixture was centrifuged at 12,000 × *g* for 15 min and the supernatant was transferred to a new tube. The protein extract was applied to a Hi-Load 16/600 Superdex 200 pg column (GE Healthcare Life Sciences, UK) equilibrated with potassium phosphate buffer. The flow rate was set at 1.0 mL/min. The eluate was collected at 3.5-min intervals, and fractions were analysed by SDS-PAGE using 11% polyacrylamide gels^25^. Proteins were stained with Coomassie Brilliant Blue R-250. For western blotting analysis, the separated proteins were electrophoretically transferred to a nitrocellulose membrane (0.45 μm; GE Healthcare Life Sciences), and recombinant proteins were detected with rabbit-derived anti-serum against the A3B4 subunit followed by goat anti-rabbit IgG-alkaline phosphatase conjugate (Promega, Fitchburg, WI, US). Protein concentrations were determined using a Protein Assay Rapid Kit (Wako, Osaka, Japan) with bovine serum albumin (BSA) used as the standard.

### Transmission electron microscopy

Transmission microscopy was carried out according to previously described methods^22^. Briefly, dry mature seeds were cut into sections and fixed for 2 h in a 4% (v/v) formaldehyde, 0.05% (v/v) glutaraldehyde solution at 4 °C. Tissue sections were washed with a sodium phosphate buffer (100 mM, pH 7.2), dehydrated in a graded ethanol series, and embedded in LR White resin (London Resin, UK). Ultrathin sections were cut using a glass knife and placed on formvar/carbon-coated grids. The sections were blocked with 1% (w/v) BSA-PBS and then incubated with anti-A3B4 subunit serum in 1% (w/v) BSA-PBS. The sections were washed and then incubated with goat anti-rabbit IgG conjugated to 15-nm gold particles (H + L, Auro Probe EM; GE Healthcare Life Sciences) in 1% (w/v) BSA-PBS. After washing, the sections were stained with 4% (w/v) uranyl acetate, and incubated with 80 mM lead nitrate. The grids were examined and photographed using a transmission electron microscope (model H-7100; Hitachi, Tokyo, Japan).

## Results

### Soybean VSR genes

**Figure 1:**
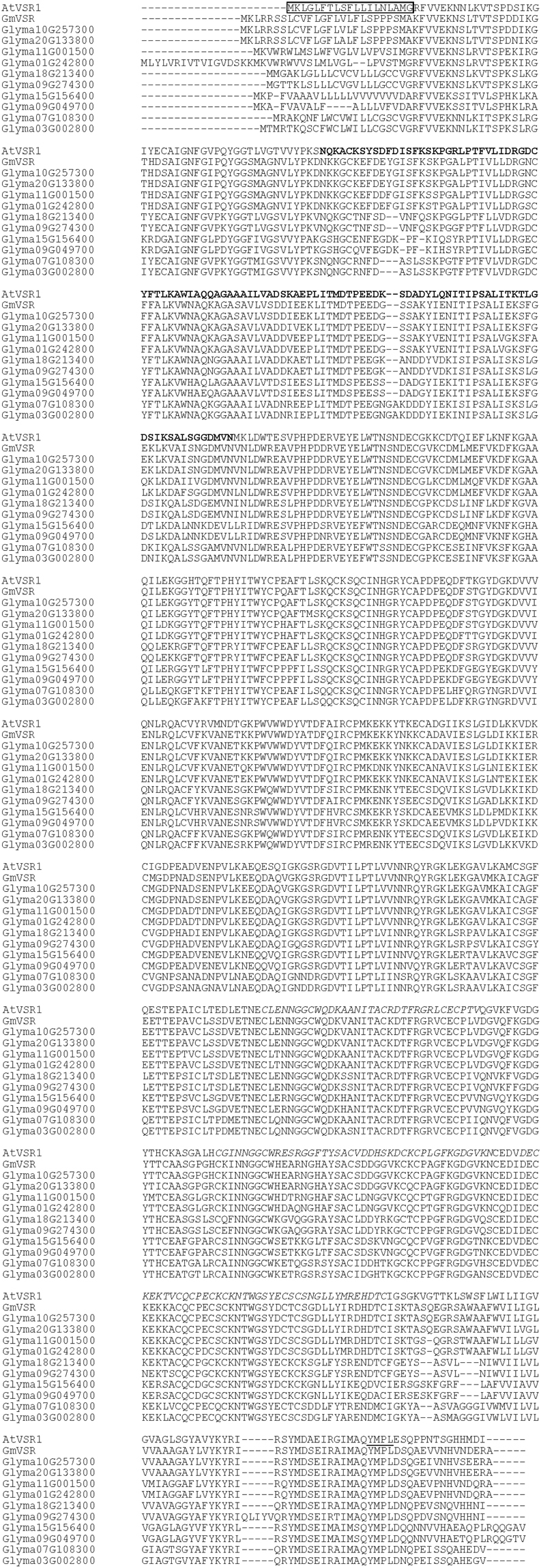
Multiple alignment of amino acid sequences of soybean VSRs and AtVSR1. Genes of soybean VSRs were extracted from Phytozome (https://phytozome.jgi.doe.gov/pz/portal.html). Multiple alignment was performed using Clustal Omega (https://www.ebi.ac.uk/Tools/msa/clustalo/). Sequences of a signal peptide, protease-associated domain, EGF-like motifs, and tyrosine sorting/internalization motif of AtVSR1 (At3g52850) are indicated by boxed, bold, italic and underlined letters, respectively.

A blast search in NCBI (https://blast.ncbi.nlm.nih.gov/Blast.cgi) indicated that the deduced amino acid sequence of the GmVSR cloned in the present study was almost identical (99%) to that of the VSR 1-like protein from *Glycine max* (accession no. XP_003536576). GmVSR shares a high amino acid identity of 85% with BP-80, which is a pea vacuolar sorting receptor^11,12^. Furthermore, we searched for VSR genes in the soybean database by using Arabidopsis VSR sequences as queries for Blast searches in the Phytozome (https://phytozome.jgi.doe.gov/pz/portal.html) and compared GmVSR with VSR genes in the soybean database (Fig. 1). Ten soybean VSR proteins were found, which share amino acid sequence identities from 57% to 99%. Expression data in the Phytozome indicates that the genes Glyma01g242800, Glyma11g001500, Glyma10g257300, Glyma20g133800, Glyma18g213400, and Glyma09g274300 are highly expressed in seeds. Among the ten soybean VSRs, the GmVSR cloned in the present study was most similar to Glyma10g257300, at 99% identity. Similar to the VSRs from other plants, GmVSR has a protease-associated (PA) domain and EGF-like motifs^10,26^. A tyrosine sorting/internalization motif (YXXΦ motif) is also present in the cytosolic tail, which interacts with the AP adaptin complex in mammalian cells, yeast, and *Arabidopsis*^27‒29^.

### Interaction of the recombinant GmVSR luminal domain with the glycinin subunits

To examine the ligand-binding mechanism of GmVSR, we expressed GmVSR(LU) in insect cells employing a baculovirus expression system. The expressed protein was purified using chelating and gel filtration columns. The N-terminal amino acid sequence of GmVSR(LU) is KFVVEKN. This corresponds to the putative N-terminal sequence of GmVSR(LU) determined using Signal P software (http://www.cbs.dtu.dk/services/SignalP) analysis, which searches for signal sequences and their cleavage sites^30^. On the basis of these results, we confirmed that the signal peptide of recombinant GmVSR(LU) is correctly processed in insect cells.

**Figure 2:**
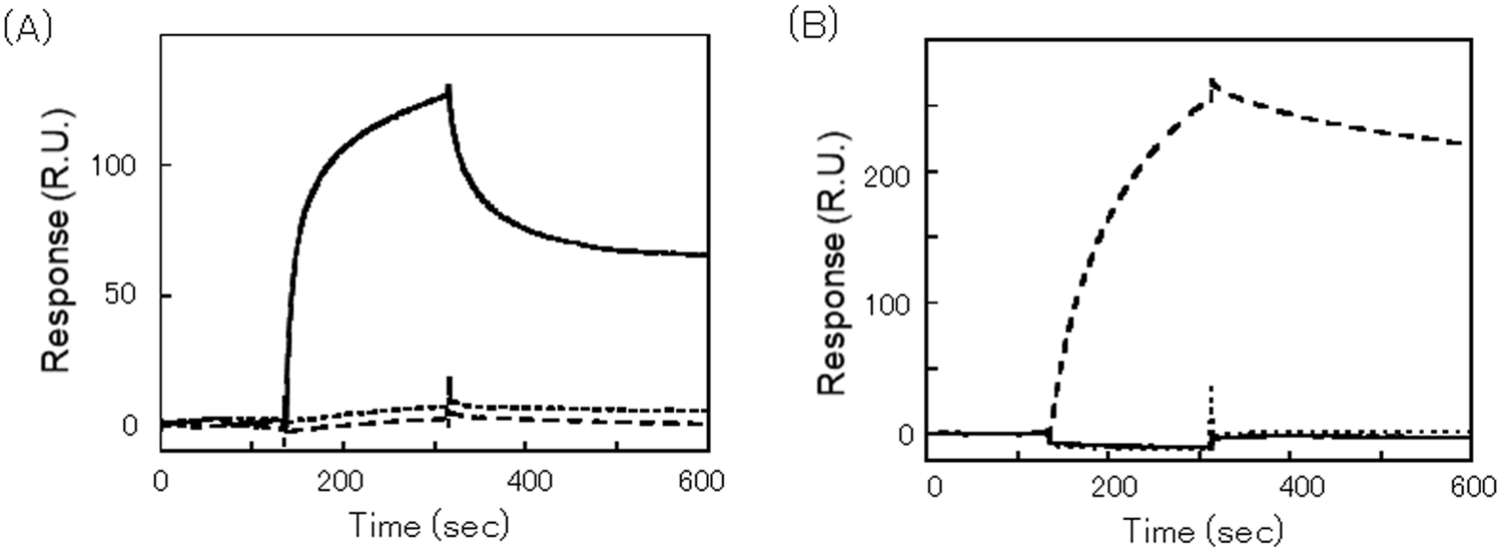
Interaction of the recombinant GmVSR luminal domain with the glycinin subunits, as determined by surface plasmon resonance. Purified proteins were injected onto GmVSR(LU) immobilized on a CM5 sensor chip for 180S, and then eluted with a salt buffer for 180S at a flow rate of 30 μL/min. (**A**) GST + A1aB1bCT10 (bold line), GST + A1aB1bCT10 + 6 G (dashed line), and Control (dotted line). (**B**) A1aB1b (dashed line), A1aB1b + 6 G (bold line), and A3B4 (dotted line).

We then examined the interaction between GmVSR(LU) and the VSDs of the A1aB1b subunit of glycinin. To investigate the interaction between receptors and glycinin in detail, the affinities of the interactions were analysed in real time by surface plasmon resonance (Fig. 2). GmVSR(LU) was immobilized on a sensor chip surface, and either GST alone, or GST fused with the C-terminal 10 amino acids (PQESQKRAVA) of A1aB1b (GST-A1aB1bCT10) were injected over the sensor chip surface. Furthermore, a sequence of six glycine residues added to GST-A1aB1bCT10 (GST-A1aB1bCT10 + 6 G) was analysed, because the vacuolar sorting function of ctVSDs is blocked by the addition of contiguous Gly residues^5,9^. GST-A1aB1bCT10 bound significantly to the GmVSR(LU) (Fig. 2A), with a dissociation constant (*K*_*D*_) of 98 nM, whereas GST and GST-A1aB1bCT10 + 6 G rarely bound to GmVSR(LU). This indicates that GmVSR has a high affinity, which is sufficient for the C-terminal 10 amino acids of the A1aB1b subunit to function as a receptor. Binding between GmVSR(LU) and GST-A1aB1bCT10 was not observed in the absence of Ca^2+^, indicating that GmVSR binding is modulated by Ca^2+^, similarly to pumpkin VSR (PV72)^31^. In contrast, GST-A1aB1bCT10 + 6 G bound weakly to GmVSR(LU).

Previously, we showed that the inhibition of A1aB1b subunit ctVSD function did not abolish the vacuolar sorting of the A1aB1b subunit completely, indicating that the A1aB1b subunit has a VSD reminiscent of psVSD as well as a ctVSD^9^. Although the three-dimensional structures of the A1aB1b and A3B4 subunits are very similar each other, the sequence corresponding to the ctVSD of the A1aB1b subunit (PQESQKRAVA) is not present in the A3B4 subunit^32,33^. Next, we investigated whether GmVSR binds to recombinant A1aB1b and A3B4 subunits that show the correct folding^21^. Because glycinin has a low solubility under low ionic strength, the surface plasmon resonance was measured in a high salt buffer (20 mM HEPES, pH 7.0, 0.4 M NaCl, 1 mM EDTA, 0.4% CHAPS, and 1 mM CaCl_2_). The A1aB1b subunit bound strongly to GmVSR, whereas the A3B4 subunit did not bind under our experimental conditions (Fig. 2B). The addition of six glycine residues to the C terminus of the A1aB1b subunit (A1aB1b + 6 G) diminished the response, and in the absence of Ca^2+^, the A1aB1b subunit could not bind GmVSR(LU) (data not shown). These observations indicate that GmVSR can interact with the A1aB1b subunit via its C-terminal peptides, but not with the A3B4 subunit.

### Vacuolar sorting of the A3B4 subunit in transgenic soybean seed

**Figure 3:**
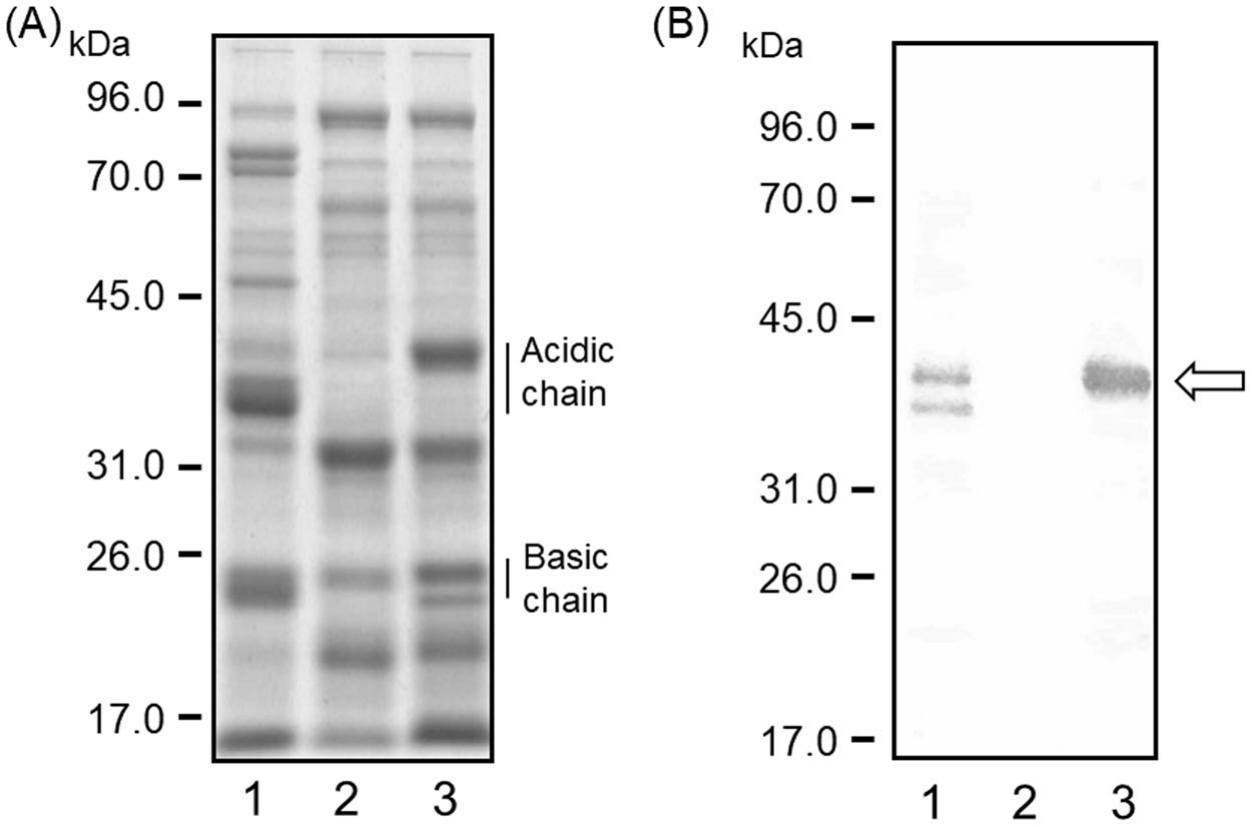
Accumulation of the A3B4 subunit in transgenic soybean seeds. SDS-PAGE and western blotting of total proteins in the control and transgenic soybean seeds accumulating the A3B4 subunit. (**A**) Total proteins were extracted from soybean seeds using SDS buffer. Proteins (12 μg) were loaded onto a 12% SDS-PAGE gel. 1, the normal cultivar (cv. Jack); 2, the JQ line; 3, transgenic soybean. (**B**) Soybean seed extracts were analysed by western blotting using an anti-A3B4 antibody. 1, the normal cultivar (cv. Jack) accumulating glycinin and β-conglynin; 2, the JQ line; 3, transgenic soybean. Arrow indicates the band corresponding to an acidic chain of A3B4 subunit. Anti-A3B4 antibody was cross-reacted with acidic chains of glycinin group I (A1a, A1b, and A2) as a second band in lane 1.

Although the QF2 breeding line lacks all subunits of the major seed storage proteins glycinin and β-conglycinin, the embryogenic response is insufficient to allow efficient transformation^34^. The JQ soybean breeding line, which lacks both glycinin and β-conglycinin and has high transformation efficiency, is generated by backcrossing breeding lines from QF2 with a normal cultivar, Jack, in which somatic embryogenesis and plant regeneration are efficient^18^. To examine sorting of the A3B4 subunit in transgenic soybean, we developed a transgenic soybean accumulating the A3B4 subunit by using the JQ soybean breeding line (Fig. 3). The JQ line also has the potential to accumulate high levels of foreign seed storage proteins^22^. The A3B4 subunit accumulated in transgenic soybean seeds in three independent transgenic lines. The post-translational-processed mature form of the A3B4 subunit, composed of acidic and basic chains, accumulated in transgenic soybean seeds. An anti-A3B4 antibody mainly detected an acidic chain of the A3B4 subunit in transgenic soybean seeds, whereas no detectable band was observed in the seeds from the JQ lines (Fig. 3B). There appears to be a substantial increase in the accumulation of several other proteins in transgenic soybean seeds. Previous reports have indicated that soybean seeds have a proteome rebalancing mechanism^35^. The accumulation of the A3B4 subunit in transgenic soybean might affect the protein profiles in seeds.

**Figure 4:**
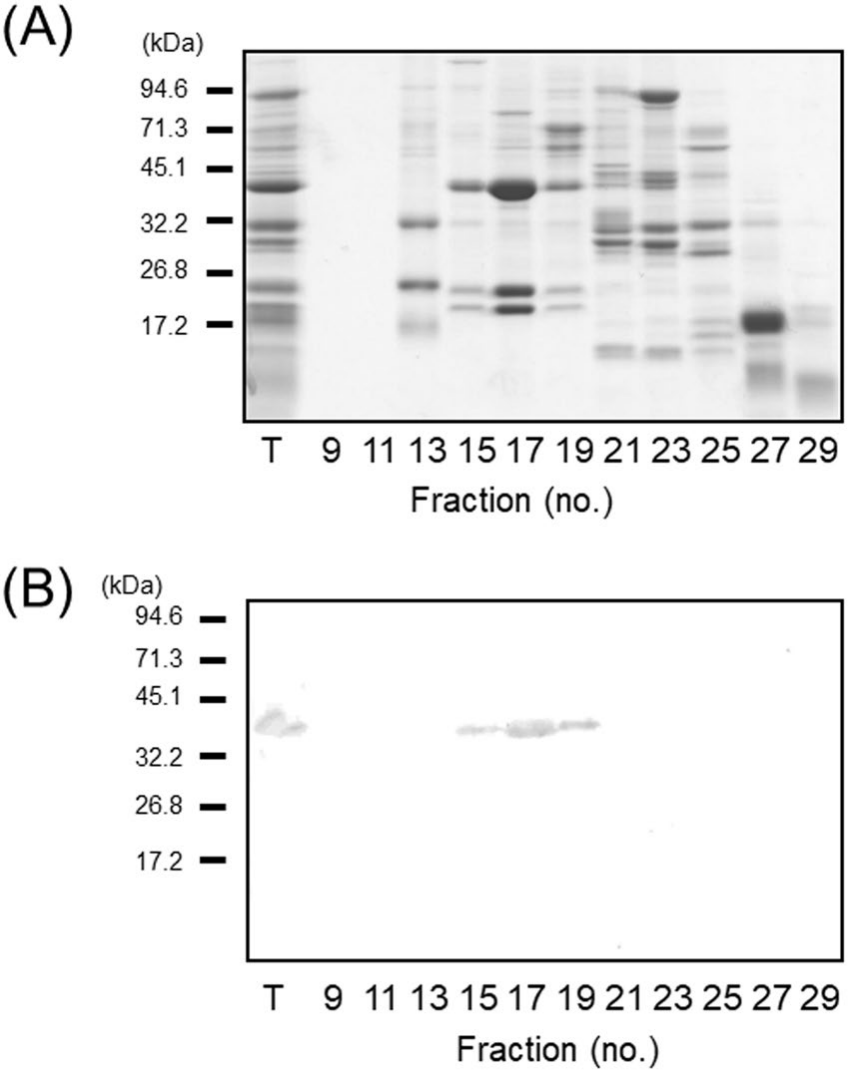
Self-assembly of the A3B4 subunit in transgenic soybean seeds, as determined by gel-filtration chromatography. (**A**) Gel-filtration elution profile of the soluble fractions using a Hi-Load 16/600 Sephacryl 200 pg column operated at 3.5 min/fraction and a flow rate of 1 mL/min. Under identical conditions, blue dextran (2000 kDa), thyroglobulin (669 kDa), ferritin (440 kDa), aldolase (158 kDa), conalbumin (75 kDa), and ovalbumin (44 kDa) as molecular mass markers were detected in fractions 12, 15, 17, 20, 22, and 24, respectively. (**B**) Western blotting of fractions obtained by gel-filtration chromatography using an anti-A3B4 antibody.

Previously, using gel-filtration chromatography we showed that the A1aB1b subunit forms the hexamer in transgenic soybean^22^. Therefore, we examined the self-assembly of the A3B4 subunit in the soluble fraction using gel-filtration chromatography (Fig. 4). A single peak containing the A3B4 subunit was detected at approximately 60 min. The fractions for the peak corresponded to the size of the hexamer form of A3B4 (approximate molecular weight of 320 kDa) by comparison with protein molecular weight standards. This suggests that the A3B4 subunit self-assembled into its mature form.

**Figure 5:**
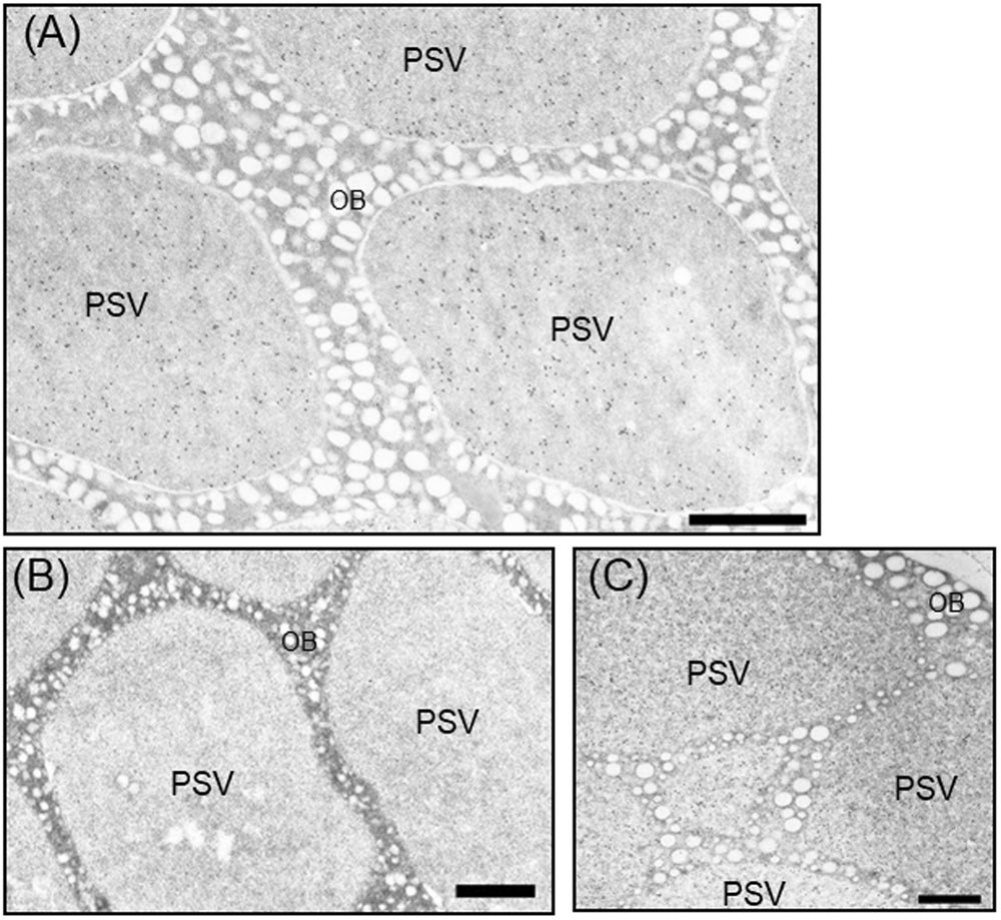
Intracellular localization of the A3B4 subunit in transgenic soybean seeds observed by immunoelectron microscopy. Immunoelectron microscopy of mature soybean seeds treated with the anti-A3B4 subunit antibody. (**A**) Transgenic soybean accumulating the A3B4 subunit, (**B**) the JQ line, (**C**) the normal cultivar (cv. Jack) accumulating glycinin and β-conglynin. PSV, protein storage vacuole; OB, oil body. Scale bar = 1.0 µm.

We assessed the localization of the A3B4 subunit by immuno-electron microscopy. In transgenic soybean seeds, the A3B4 subunit was sorted into protein storage vacuoles, as indicated by the deposition of gold particles conjugated with anti-A3B4 antibody, whereas gold particles were rarely observed in seed cells under the same conditions in the seeds of JQ lines (Fig. 5). We rarely observed the protein bodies originated from the ER in the JQ line and transgenic soybean seeds. These suggest the existence of a protein storage vacuolar sorting mechanism in soybean seeds that functions independently of the known vacuolar sorting receptors in seeds.

## Discussion

In this study, the luminal domain of soybean VSR was recombinantly prepared using an insect cell system. Surface plasmon resonance experiments demonstrated binding of the VSR to the 10 C-terminal amino acids of the glycinin A1aB1b subunit, whereas we detected no binding to the A3B4 subunit lacking this C-terminal sequence. A3B4 was then expressed in a soybean line lacking endogenous glycinin and β-conglycinin and assembly and deposition in PSVs were confirmed.

In *A*. *thaliana*, there are seven genes for VSR (AtVSR1–7)^14^, which are divided into three classes: class 1 (*AtVSR1* and *AtVSR2*), class 2 (*AtVSR3* and *AtVSR4*), and class 3 (*AtVSR5*, *AtVSR6*, and *AtVSR7*). The members of class 1 and 2 are functionally closely related because AtVSR1, AtVSR3, and AtVSR4 are involved in trafficking to the vacuoles^14,36,37^. The PA domains of AtVSR1, AtVSR3, and AtVSR4 are more similar to the pea vacuolar sorting receptor BP-80 than to AtVSR5, AtVSR6, and AtVSR7, suggesting that similarities in the PA domain are related to ligand-binding specificity^11^. AtVSR6 and AtVSR7 are highly expressed in the roots and may be associated with a defence response^38^. Expression data indicate that the genes Glyma01g242800, Glyma11g001500, Glyma10g257300, Glyma20g133800, Glyma18g213400, and Glyma09g274300 are highly expressed in seeds. The PA domains of the soybean VSRs expressed in seeds exhibit higher amino acid identities with AtVSR1, AtVSR3, and AtVSR4 (59–82%) than with AtVSR6 and AtVSR7 (49–52%). This may indicate that a VSR subclass with a similar function to that of AtVSR1, AtVSR3, and AtVSR4 is expressed in soybean seeds.

Previously, we analysed the VSDs of A1aB1b and A3B4 subunits using transient expression assays with fluorescent proteins. On the basis of these assays, we indicated that the C-terminal region of the A1aB1b subunit can function as a ctVSD and suggested that the A3B4 and the A1aB1b subunits probably possess a VSD, such as a psVSD, although not a ctVSD or ssVSD. In the present study, we found that the VSR protein of soybean appears to be involved in sorting via a ctVSD. However, the A1aB1b subunit fused with an additional six glycine residues at the C terminus (A1aB1b + 6 G), and the A3B4 subunit did not bind to the VSR expressed in soybean seeds. These observations suggest that a receptor other than the known VSRs in seeds might be involved in the PSV sorting by a VSD, such as a psVSD.

There are two pathways by which seed storage proteins are transported to the PSVs in soybean: one via the Golgi apparatus and the other directly from the ER to the PSVs^39^. Although the protein bodies originated from the ER appear to be involved in the direct pathway from the ER to the PSV, we rarely observed the protein bodies originated from the ER in the JQ line and transgenic soybean seeds. Previously, electron microscopic analysis of developing soybean cotyledons of mutant lines with storage protein composition different from that of the wild type showed that the protein bodies originated from the ER were hardly observed in the mutant lines lacking 11S group I subunit (A1aB1b, A1bB2, and A2B1a)^36^. These observations indicate that the A3B4 subunit is transported to the PSVs mainly by a pathway via the Golgi apparatus. Recently, it has been reported that VSRs are required for the transport of ligands from the ER and the Golgi to the trans-Golgi network^13^. These reports, together with the findings of the present study, indicate that a novel receptor for the transport of ligands in these steps might be present in soybean seeds. Further studies are required to elucidate the receptors for sorting and VSDs of seed storage proteins in soybean seeds.

## References

[1] Vitale A, Hinz G (2005). Sorting of proteins to storage vacuoles: how many mechanisms?. Trends Plant Sci..

[2] Saalbach G, Rosso M, Schumann U (1996). The vacuolar targeting signal of the 2S albumin from Brazil nut resides at the C terminus and involves the C-terminal propeptide as an essential element. Plant Physiol..

[3] Jolliffe NA (2004). Transport of ricin and 2S albumin precursors to the storage vacuoles of Ricinus communis endosperm involves the Golgi and VSR-like receptors. Plant J..

[4] Frigerio L, de Virgilio M, Prada A, Faoro F, Vitale A (1998). Sorting of phaseolin to the vacuole is saturable and requires a short C-terminal peptide. Plant Cell.

[5] Nishizawa K (2003). A C-terminal sequence of soybean β-conglycinin α′ subunit acts as a vacuolar sorting determinant in seed cells. Plant J..

[6] Nishizawa K, Maruyama N, Satoh R, Higasa T, Utsumi S (2004). A vacuolar sorting determinant of soybean β-conglycinin β subunit resides in a C-terminal sequence. Plant Sci..

[7] Saalbach G (1991). Different legumin protein domains act as vacuolar targeting signals. Plant Cell.

[8] Hegedus DD (2015). Multiple internal sorting determinants can contribute to the trafficking of cruciferin to protein storage vacuoles. Plant Mol. Biol..

[9] Maruyama N (2006). Multiple vacuolar sorting determinants exist in soybean 11S globulin. Plant Cell.

[10] Ahmed SU, Bar-Peled M, Raikhel NV (1997). Cloning and subcellular location of an Arabidopsis receptor-like protein that shares common features with protein-sorting receptors of eukaryotic cells. Plant Physiol..

[11] Cao X, Rogers SW, Butler J, Beevers L, Rogers JC (2000). Structure requirements for ligand binding by a probable plant vacuolar sorting receptor. Plant Cell.

[12] Kirsch T, Paris N, Butler JM, Beevers L, Rogers JC (1994). Purification and initial characterization of a potential plant vacuolar targeting receptor. Proc. Natl. Acad. Sci. USA.

[13] Künzl F, Früholz S, Fäßler F, Li B, Pimpl P (2016). Receptor-mediated sorting of soluble vacuolar proteins ends at the trans-Golgi network/early endosome. Nat. Plants.

[14] Robinson DG, Neuhaus JM (2016). Receptor-mediated sorting of soluble vacuolar proteins: myths, facts, and a new model. J. Exp. Bot..

[15] Shimada T (2003). Vacuolar sorting receptor for seed storage protein in Arabidopsis thaliana. Proc. Natl. Acad. Sci. USA.

[16] Fuji K (2007). Arabidopsis vacuolar sorting mutants (green fluorescent seed) can be identified efficiently by secretion of vacuole-targeted green fluorescent protein in their seeds. Plant Cell.

[17] Utsumi S (1992). Plant food protein engineering. Adv. Food Nutr. Res..

[18] Kita Y, Nishizawa K, Takahashi M, Kitayama M, Ishimoto M (2007). Genetic improvement of the somatic embryogenesis and regeneration in soybean and transformation of the improved breeding lines. Plant Cell Rep..

[19] Maruyama N (2015). Preliminary X-ray analysis of the binding domain of the soybean vacuolar sorting receptor complexed with a sorting determinant of a seed storage protein. Acta Crystallogr. F Struct. Biol. Commun..

[20] Morimoto S, Tomohiro T, Maruyama N, Hatanaka Y (2013). Photoaffinity casting of a coumarin flag for rapid identification of ligand-binding sites within protein. Chem. Commun..

[21] Prak K (2005). Structure-function relationships of soybean proglycinins at subunit levels. J. Agric. Food Chem..

[22] Maruyama N (2014). Stable accumulation of seed storage proteins containing vaccine peptides in transgenic soybean seeds. J. Biosci. Bioeng..

[23] Goosens A, Dillen W, De Clercq J, Van Montagu M, Angenon G (1999). The arcelin-5 gene of *Phaseolus vulgaris* directs high seed-specific expression in transgenic *Phaseolus acutifolius* and Arabidopsis plants. Plant Physiol..

[24] Khalafalla MM, Mizanur RS, Nakamoto Y, Wakasa K, Ishimoto M (2005). Optimization of particle bombardment condition by monitoring of transient sGFP(S65T) expression in transformed soybean. Breed. Sci..

[25] Laemmli UK (1970). Cleavage of structural proteins during the assembly of the head of bacteriophage T4. Nature.

[26] Luo X, Hofmann K (2001). The protease-associated domain: a homology domain associated with multiple classes of proteases. Trends Biochem. Sci..

[27] Ohno H (1995). Interaction of tyrosine-based sorting signals with clathrin-associated proteins. Science.

[28] Marks MS, Woodruff L, Ohno H, Bonifacino JS (1996). Protein targeting by tyrosine-and di-leucine-based signals: evidence for distinct saturable components. J. Cell Biol..

[29] Fuji K (2016). The Adaptor Complex AP-4 Regulates Vacuolar Protein Sorting at the trans-Golgi Network by Interacting with VACUOLAR SORTING RECEPTOR1. Plant Physiol..

[30] Petersen TN, Brunak S, von Heijne G, Nielsen H (2011). SignalP 4.0: discriminating signal peptides from transmembrane regions. Nat. Methods.

[31] Watanabe E, Shimada T, Kuroyanagi M, Nishimura M, Hara-Nishimura I (2002). Calcium-mediated association of a putative vacuolar sorting receptor PV72 with a propeptide of 2S albumin. J. Biol. Chem..

[32] Adachi M, Takenaka Y, Gidamis AB, Mikami B, Utsumi S (2001). Crystal structure of soybean proglycinin A1aB1b homotrimer. J. Mol. Biol..

[33] Adachi M (2003). Crystal structure of soybean 11S globulin: glycinin A3B4 homohexamer. Proc. Natl. Acad. Sci. USA.

[34] Takahashi M (2003). Accumulation of high levels of free amino acids in soybean seeds through integration of mutations conferring seed protein deficiency. Planta.

[35] Schmidt MA, Herman EM (2008). Proteome rebalancing in soybean seeds can be exploited to enhance foreign protein accumulation. Plant Biotechnol. J..

[36] Zouhar J (2010). Functional specialization within the vacuolar sorting receptor family: VSR1, VSR3 and VSR4 sort vacuolar storage cargo in seeds and vegetative tissues. Plant J..

[37] Lee Y (2013). Functional identification of sorting receptors involved in trafficking of soluble lytic vacuolar proteins in vegetative cells of *Arabidopsis*. Plant Physiol.

[38] Wang D (2005). Induction of protein secretory pathway is required for systemic acquired resistance. Science.

[39] Mori T (2004). The composition of newly synthesized proteins in the endoplasmic reticulum determines the transport pathways of soybean seed storage proteins. Plant J..

